# Early Sociocognitive Development Among Spanish-Speaking Participants: A Preliminary Study Using the Early Social Cognition Inventory (ESCI)

**DOI:** 10.3390/children13070932

**Published:** 2026-07-16

**Authors:** Kostadin Kostov, Yurena Alonso-Esteban, Francisco Alcantud-Marín

**Affiliations:** 1 Department of Developmental and Educational Psychology, University of Valencia, 46010 Valencia, Spain; koshkos@alumni.uv.es (K.K.); francisco.alcantud@uv.es (F.A.-M.); 2 Department of Developmental and Educational Psychology, University of La Laguna, 38200 Santa Cruz de Tenerife, Spain

**Keywords:** sociocognitive development, adaptation, psychometrics, ESCI, early measurement

## Abstract

Background: The early development of sociocognitive skills is linked to, and serves as a precursor for, the development of communication, language, and socialisation. Some of the most prevalent neurodevelopmental disorders, such as autism, are characterised by difficulties in communication and social interaction. Identifying delays in these skills could enable us to detect an earlier warning sign. Method: The original version of the Early Social Cognition Inventory (ESCI), a 21-item parent-reported questionnaire, was translated and culturally and linguistically adapted for use in Spain. Participants: The sample included 113 children from nursery schools and 19 children from an early intervention centre, aged between 18 and 60 months, from 10 early childhood centres in Valencia (Spain). Parents completed the ESCI and the Social Reactivity Scale, Preschool Second Edition (SRS-2 P). The children were assessed using additional tests, including the Early Sociocognitive Battery (ESB) and the Peabody Picture Vocabulary Test III (PPVT). Results: Internal consistency (α = 0.78, *p* < 0.001) and the correlation between criterion measures (*p* < 0.001) indicate that the ESCI measures sociocognitive development. Furthermore, the results regarding diagnostic ability, assessed using the AUC (area under the curve) values from the ROC analysis, are promising (AUC = 0.94; *p* < 0.001 for Autism Spectrum Disorders), although further research is needed given the sample size. Conclusions: The results of the ESCI pilot study indicate that it is a suitable tool for assessing early sociocognitive development in Spain, although further research with larger samples and longitudinal studies is required.

## 1. Introduction

The development of social cognition refers to the acquisition of key skills that enable children to interact meaningfully with their environment, caregivers, and peers [1,2,3]. Early sociocognitive skills, including social orientation, joint attention and symbolisation, begin to develop in the first few years of life [4], and influence the development of social interaction and the emergence of language [5,6].

As these early sociocognitive skills develop, meta-representational skills (Theory of Mind—ToM) will subsequently emerge. This ability allows us to infer other people’s mental states—such as beliefs, desires or intentions—and to use this information to understand, explain and predict their behaviour [7]. From this perspective, understanding the minds of others enables individuals to anticipate the actions of others and adapt their own behaviour within the social environment. In this process of understanding the intentions and actions of others, children begin to use various social cues from their environment. For this reason, ToM has been regarded as a fundamental component of sociocognitive development and a key element in the acquisition of social and communicative skills during childhood.

Early deficits in sociocognitive skills, such as reduced social gaze, delayed joint attention, and limited pragmatic language, are often early indicators of a neurodevelopmental disorder (NDD). For example, children with autism display atypical patterns of eye contact and social reciprocity as early as six months [8,9]. These communication difficulties can create a vicious circle, in which delays in sociocognitive development further isolate the individual and limit opportunities for meaningful interaction. Sociocognitive deficits are common in both autistic children and adolescents and, to a lesser extent, in adults [10,11,12].

The importance of the clinical assessment of sociocognitive development has been recognised through its inclusion in the DSM-5 [13,14], linking the deficit to various neurodevelopmental disorders (NDDs) [15,16]. In particular, the role of social cognition deficits in autism spectrum disorders has been the focus of numerous studies [17,18,19], both for their predictive value [20,21] and in terms of planning early intervention [22,23].

For this reason, early assessment of sociocognitive skills is essential for the early detection of potential developmental difficulties. However, assessing sociocognitive skills through observation requires multiple sessions and the use of complex tools, making it a time-consuming and costly process. In this regard, tools for assessing sociocognitive skills in early childhood measure the development of social understanding, joint attention and symbolisation through direct applications such as the Early Sociocognitive Battery (ESB) [24], delivered via tablets such as the Early Years Toolbox (EYT) [25] or reported by parents and caregivers as the Global Scales for Early Development (GSED), [26], CSUS (Children’s Social Understanding Scale); [27], el ToMI (Theory of Mind Inventory), [28]. Unfortunately, there are very few tools available for assessing sociocognitive skills in children under the age of two. In this regard, the ESCI (Early Social Cognition Inventory) [29] particularly caught our attention due to the age range for which it is designed and its predictive value regarding ASD [30]. In this article, we present the preliminary results of the translation and adaptation of the ESCI for the Spanish population, examining its reliability, concurrent validity, and discriminant validity.

## 2. Materials and Methods

### 2.1. Procedure

The data collection was carried out as part of the project to adapt the Early Sociocognitive Battery for use in Spain (ESB) [24]. A call for participation was issued to public and private nursery schools in the Valencia metropolitan area; a total of ten schools took part. The schools that expressed an interest received a report containing the results of the assessment tests as a token of appreciation. At each school, parents were contacted via the school management or the school guidance service to obtain informed consent, in accordance with the guidelines approved at the meeting of 3 November 2022 by the University of Valencia’s Ethics Committee for Research Involving Human Subjects (UV-1NV_ETICA-2053373). Data collection began in January and ended in June 2024.

Once contact had been established with the school, the required number of copies of the information letter, the informed consent forms, the demographic data, the instructions document, and the ESCI were sent to the school. Parents who agreed to have their children assessed completed all the information and placed it in an envelope, which they sealed themselves to ensure the confidentiality of the data. Similarly, the results reports were delivered to the school in a sealed envelope addressed to the parents of the child assessed.

### 2.2. Participants

Participants in the ESB adaptation project were invited to complete the ESCI questionnaire as well. Of the nearly 350 participants in that study, we received 113 responses from children attending nursery schools or childcare centres in the Valencia metropolitan area. In addition, we contacted a Child Development and Early Intervention Centre (CDIAT), which selected 19 cases for us diagnosed with Language Delay (LD) and Autism Spectrum Disorders (ASD). Although the original version of the ESCI was designed for use with young children up to 47 months [29], 31 children aged between 48 and 60 months took part. We considered whether or not to include them in this study, and, given its preliminary nature, we deemed it appropriate to include them in order to determine the upper age limit for its use. Consequently, for this preliminary study, a convenience sample was drawn comprising a group of children with no known (clinical) diagnosis, comprising 113 children (58.04% boys and 41.96% girls), and a second group comprising 19 children of a similar age who had been diagnosed with neurodevelopmental disorders, as shown in Table 1.

With regard to the socioeconomic and cultural circumstances of the families, it should be noted that the sample comprised 92.56% Spanish nationals and 7.44% of other nationalities. From an economic perspective, 40.75% of families had an annual income of over €30,000. All parents had sufficient reading skills to complete the ESCI questionnaire, and 66.50% of participants had completed secondary or higher education.

### 2.3. Measures

#### 2.3.1. Early Social Cognition Inventory (ESCI)

The ESCI assesses social cognition in children aged between 4 and 39 months using a 21-item questionnaire completed by parents [29]. According to the authors, this measure demonstrates a high level of internal reliability, a coherent factor structure, test stability, inter-rater reliability, and significant longitudinal stability in children aged between 4 and 39 months. Sadeghi et al. [30] conducted a study on the ESCI with the aim of determining its validity for the detection of autism, obtaining a correlation with the MCHAT of 0.786 (*p* < 0.001), and a reliability and internal consistency (Kappa 20) of 0.85. As for construct validity, exploratory factor analysis of the sample of autistic children revealed the existence of two factors (joint attention and understanding of beliefs and emotions), reducing the scale to just 17 items, which they named the ESCI-AV.

We contacted the authors and began the translation and adaptation process following the guidelines for translation and adaptation of Psychological Assessment instruments [31,32]. Firstly, the translation was carried out by two bilingual psychologists. The differences were compared and contrasted, resulting in a unified Spanish version. In a second phase, a back-translation into English was carried out. The 21 items in the questionnaire were presented to a group of three parents to check whether the items were conceptually understood. The proposed changes mainly concerned the examples given. For item 1, it was proposed to include the following example: Item 1: Does your child follow where you look in order to look at the same thing as you? For example, if you look up at the sky as an aeroplane flies past, does your child follow your gaze towards the aeroplane? In other items, the examples were replaced or culturally adapted; for example, in item 5: ‘Is your child aware of other people’s perspectives?’ The original example was complex and was replaced with: ‘Does your child understand that they can see an object that you or another person cannot see from a different angle?’

The final questionnaire had the same structure as the original version, although it was decided to include dichotomous responses (YES/NO) with a third option such as ‘I don’t know/I haven’t observed this’. We included the third response option because this was a preliminary study. Given that the items cover behaviours typical of children aged between 4 and 47 months, we felt that some parents might skew their responses when faced with a pure dichotomy, as they might perceive that they were assessing their child’s abilities. The Supplementary Material includes a table showing the response frequencies for each option across the 21 items. In order to construct a total score consistent with the trait being measured, affirmative responses were scored as 2 and the rest as 1 following the model proposed by Sadeghi et al. [30]. Thus, the score ranges from 21 to 42 points, indicating that a higher score implies greater sociocognitive development. The total score was calculated as the sum of the responses to each item, and it was found that the variance of the total score remained unchanged when the scoring method was altered.

#### 2.3.2. Social Responsiveness Scale, 2nd Edition, Preschool Version

The SRS-2 assesses the severity of the most common symptoms of autism and is the result of improvements made to previous versions [33]. The current version comprises a family of questionnaires organised by age group: the preschool version (30–54 months), the school-age version (4–18 years), the young adults and adults version (19–89 years), and the self-report version for adults. In all its forms, the SRS-2 consists of 65 four-point Likert-type response items (with a standardised score ranging from 0 to 3). The items have been classified into five subscales: Social Awareness (AWR), Social Cognition (COG), Social Communication (COM), Social Motivation (MOT), and Repetitive Behaviours and Restricted Interests (RRB). The SRS-2 follows the diagnostic criteria of the DSM-5-TR [14], proposing two domains. The first, Social Communication and Interaction (SCI), would be calculated by summing the first four subscales, whilst the second criterion, Restricted Interests and Repetitive Behaviours (RRB), would be assessed using the final subscale of the same name. The SRS-2 has been extensively studied across different populations and cultures, demonstrating good psychometric indicators of internal consistency, construct validity, etc. The version used in this study is the preschool version. The internal consistency of the SRS-2 Preschool ranges from 0.71 to 0.98, whilst the test–retest reliability of the US version was 0.75.

#### 2.3.3. Peabody III Picture Vocabulary Test

The Peabody III Picture Vocabulary Test [34] is a tool designed to assess linguistic listening comprehension through receptive vocabulary (for ages 2 to 90), and can be used both as a screening tool for language disorders and to complement the assessment of complex cognitive processes; it is presented in blocks of 12 items organised by age group. The test was administered in accordance with the guidelines for the start and end of the test. The internal consistency of the Spanish version (Alpha 0.93) and its concurrent validity (0.25 and 0.69) with the Kaufman-ABC scale make it an excellent measure of the level of linguistic comprehension development.

#### 2.3.4. Early Sociocognitive Battery (ESB)

The Early Sociocognitive Battery [24] consists of three subscales: Responsive Sociality (SR), Joint Attention (JA), and Symbolic Understanding (SU), and takes approximately 15 to 20 min to administer. The Spanish adaptation of this instrument has yielded very high internal consistency coefficients (SR 0.86; JA 0.95; SU 0.98; total 0.99), and test–retest reliability indicates that the battery is stable (SR 0.74; JA 0.76; SU 0.81; total 0.90), with high inter-rater reliability as well (SR 0.80; JA 0.96; SU 0.98; total 0.96).

### 2.4. Data Analysis

Descriptive analyses were performed on the total ESCI score by age, using mean, standard deviation, and graphical representations. Conventionally, Cronbach’s alpha [35] has been used as an indicator of the internal consistency of a psychometric measurement instrument [36].

Construct validity is the ability of an instrument to accurately measure the construct it is designed to measure [37]. This can be determined using either convergent validity or known clinical groups. Convergent validity refers to the degree to which scores obtained using the instrument under evaluation correlate with those obtained using other reference instruments. Regarding recognised groups, it refers to the instrument’s ability to characterise individuals from different groups [38].

The capacity to discriminate between two health conditions or diagnostic efficiency will be determined by analysing the receiver operating characteristic (ROC) curve [39]. This analysis compares the sensitivity and specificity of the tests to be evaluated (in our case, the ESCI) and determines the most appropriate cut-off scores for detecting ASD or other neurodevelopment disorders in Spanish preschool children. The area under the curve (AUC) serves as an indicator of performance, with a value of 0.50 meaning that the diagnostic tool has no discriminatory power compared to the reference standard, while a value of 1.0 means complete power; a value of 0.70 or higher indicates an adequate level of discriminatory accuracy. In addition, the Gini coefficient [40] and Youden Index [41] were calculated to help assess the accuracy of the prediction; their values range from 0 (the measure has no discriminatory power) to 1 (perfect discrimination). A GINI value is considered robust when it exceeds 0.60. Similarly, the YOUDEN Index also takes values between 0 and 1. In this case, the aim here is to determine the optimal threshold (cut-off) that maximises the effectiveness of a marker. In all calculations, the confidence interval was set at 95%. Version 29 of SPSS, licenced by the University of Valencia, was used for statistical calculations.

## 3. Results

**Figure 1:**
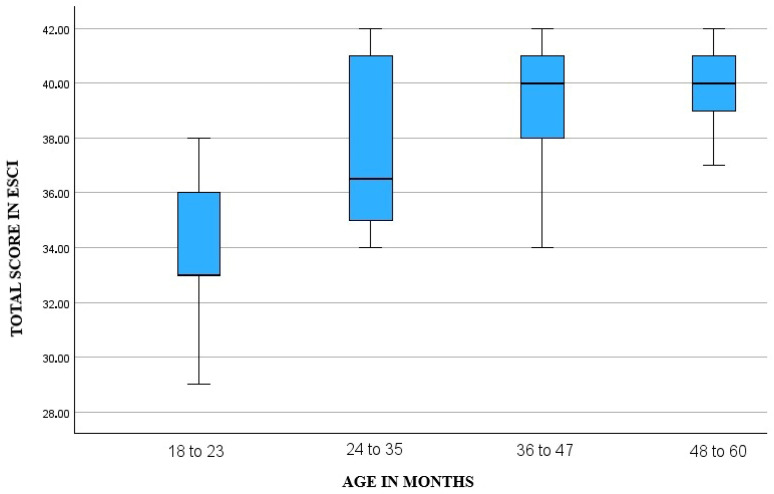
Boxplot showing the total ESCI score for the four age groups analysed. The box plot graphically displays five values: the maximum and minimum values, shown as solid lines; the first quartile (Q_1_) and third quartile (Q_3_), which define the box; and the median (Q_2_), represented by a thick horizontal line.

The Supplementary Material presents an analysis of the items. Item 3 shows zero variance (a constant response) and is therefore excluded from all calculations. Once the total ESCI score was calculated, descriptive statistics were computed, showing that the total ESCI score measures a trait whose value depends on the child’s developmental age according to Pearson’s correlation coefficient (r_xy_ = 0.67, *p* < 0.01). Table 2 presents the descriptive statistics for the four age groups and the total sample. The results of the ANOVA across the four age groups reveal differential patterns (F = 31.69, *p* < 0.001), although these analyses will need to be replicated with larger, more balanced samples. The box plot in Figure 1 illustrates the described pattern and a ceiling effect in the ESCI score corresponding to the oldest age group in the sample.

Internal consistency was calculated for the sample of typically developing children, yielding an alpha coefficient of 0.78 (*p* < 0.001); a Guttman two-half test of 0.67 (*p* < 0.001); and an intraclass correlation coefficient (ICC) of 0.78 (*p* < 0.001). With regard to concurrent validity, the correlation between the total ESCI score and the scores on the subscales and total scores of the criterion tests used was calculated (see Table 3). With regard to the Peabody score, a high and significant correlation was observed, which we interpret as meaning that the construct measured by the ESCI score assesses maturation in line with developmental age.

Furthermore, all subscales of the ESB correlate significantly with the ESCI (*p* < 0.001), both across the total sample and specifically within the sample of typically developing children. It is worth noting that the group of children with neurodevelopmental disorders attending CDIAT also showed correlations with the ESB, although in this case the values were significant (*p* < 0.05) but slightly lower. These results are reasonable given the size of the groups. Consequently, and on a preliminary basis, we can say that the ESCI, as a construct, also measures sociocognitive development. It is important to bear in mind that both PPVT and ESB correlate with age; therefore, in future studies, partial correlation should be analysed in order to account for the effect of chronological age.

Finally, the ESCI was correlated with the SRS-2, revealing significant negative correlations (*p* < 0.001), as SRS-2 scores measure symptom severity and, therefore, deviation from neurotypical development. Note that the subscale with the lowest correlation is that related to repetitive behaviours and restricted interests (RRB), which loses its significance when calculated for the group of children with neurodevelopmental disorders.

**Figure 2:**
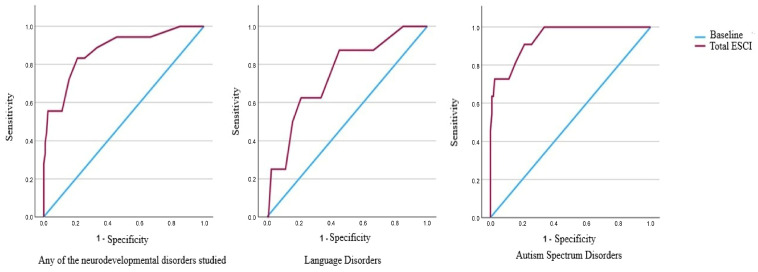
Graphical representation of the ROC curves for language disorders, autism spectrum disorders, or both.

Table 4 shows the descriptive statistics for typically developing children and the two target groups (language disorders and autism spectrum disorders). It should be noted that this is a convenience sample and that there is a significant disparity in the number of children in each category and age group. For this reason, we have chosen to perform exploratory ROC calculations on the sample as a whole, without taking the age variable into account. With regard to diagnostic performance, ROC curves were calculated for each group of neurodevelopmental disorders (language disorders, autism spectrum disorders, or both), yielding the results shown in Table 5 and Figure 2. The positive predictive values (PPV)—that is, the probability that children scoring below the respective exploratory cut-off points will actually develop a neurodevelopmental disorder (LD and ASD)—are very high (<0.95).

The data in Table 5 demonstrate that the total ESCI score performs well in distinguishing children with neurotypical development from those attending CDIAT centres due to a diagnosis of a neurodevelopmental disorder (this study considered only language disorders and autism spectrum disorders). It can be seen that the results are better when the classification is restricted to children diagnosed with ASD. Given the size and characteristics of the sample, these findings, whilst promising, should be treated with caution. In any case, the ESCI is an instrument that assesses socio-cognitive development and, as such, could identify a delay in the development of these skills in a child, which in turn may serve as a warning sign of a possible neurodevelopmental disorder.

In short, it could form part of a battery of tools for monitoring a child’s development and, on occasion, serve as a complementary tool to help determine whether it is appropriate or necessary to use a more specific screening tool.

## 4. Discussion

There are currently numerous parent-reported tools for the detection of ASD [42], most of which have a lower age limit of between 18 and 24 months and are based on the assessment of known warning signs or symptoms of autism [43,44]. To bring forward the age at which any neurodevelopmental disorder is detected, we need to put early warning systems in place. Early sociocognitive development, given its implications and links to the development of communication and language, could be one such system. In this pilot study, the age range for the ESCI was restricted to align with the age limits of the reference tests (PPVT, ESB and SRS-2) in order to verify that the construct assessed by the 21-item ESCI measures the child’s sociocognitive development. The results obtained suggest this relationship, although we should treat the results with caution. Firstly, the significant positive correlation between chronological age (measured in months) and the total ESCI score indicates that the sociocognitive skills assessed follow a developmental trajectory, as do the PPVT and ESB scores. These findings are consistent with developmental theories, which suggest that sociocognitive development progresses gradually during early childhood and forms the basis for subsequent skills. The correlations obtained between the ESCI and the reference measures used in the pilot study provide support for the construct validity of the instrument. Significant positive correlations were observed between the ESCI and the Early Sociocognitive Battery (ESB), particularly with the Symbolic Understanding and Joint Attention subscales. This finding is theoretically consistent, as these skills represent central dimensions of early sociocognitive functioning [45,46]. Similarly, the significant negative correlations found between the ESCI and the SRS-2 indicate that lower sociocognitive performance is associated with greater severity of autism-related symptoms. These findings are particularly relevant because they reinforce the idea that early sociocognitive development may serve as a marker of risk for neurodevelopmental disorders [5,47,48].

The results also showed strong correlations between the ESCI and receptive language skills, as measured using the Peabody Picture Vocabulary Test. This relationship is predictable, given the close developmental association between sociocognitive skills and language acquisition. Early joint attention, symbolic representation, and social receptivity are considered fundamental precursors of communication and language development [49]. Consequently, delays in sociocognitive functioning may negatively affect subsequent communicative competence and social adaptation.

Another finding concerns the discriminatory performance of the ESCI. Although these results must be interpreted with caution due to the size and characteristics of the sample, the results of the ROC analysis demonstrated that the instrument shows good discriminatory power in distinguishing typically developing children from those diagnosed with neurodevelopmental disorders. The strongest performance was observed in relation to autism spectrum disorder, where the area under the curve reached very high values. These results suggest that the measure of sociocognitive development could be used as a predictor of neurodevelopmental disorders, particularly ASD [6], although it will be necessary to conduct longitudinal studies with larger samples to determine the predictive value of the measure. However, the findings also indicate that sociocognitive development should not be interpreted using a single universal cut-off point. Youden’s index analyses suggest that different thresholds may be necessary depending on age. This issue is particularly important given the developmental nature of the construct measured by the ESCI. A score that may indicate a developmental risk at 18 months might be considered appropriate for development at an earlier age or insufficiently sensitive at a later age. Therefore, future studies should establish normative developmental trajectories and age-adjusted cut-off points.

An additional issue arising from the results concerns the ceiling effect observed in older children, particularly in the 48–60-month age group. Descriptive analyses suggest that many children in this group achieved scores close to the maximum possible score. Given the ceiling effect, the ESCI should be used with caution after 48 months in typically developing children. This finding may indicate that the ESCI is particularly sensitive during the early stages of development but becomes less discriminatory as sociocognitive skills consolidate with age.

### Limitations and Future Lines of Research

We must acknowledge some limitations of the present study. This is a preliminary study carried out on a convenience sample; therefore, all results should be treated with caution. Firstly, both the sample of typically developing children and the sample of children with neurodevelopmental disorders were relatively small, especially when divided into diagnostic subgroups. As this is a convenience sample and a preliminary study, the group sizes are not balanced; therefore, the results should be treated with caution pending further evidence. This limits the generalisability of the results and may have influenced the stability of some statistical indicators. Secondly, the study employed a cross-sectional design, which prevented the analysis of developmental trajectories over time. Longitudinal studies would be particularly valuable for determining the predictive validity of the ESCI and for developing age-based norms to analyse whether the early sociocognitive difficulties identified by the instrument remain stable throughout development.

Longitudinal studies are needed to assess the ESCI’s predictive power regarding subsequent neurodevelopmental outcomes. Future research should focus on expanding the clinical sample, establishing age-based normative data, and examining the factor structure. Furthermore, future research should explore the applicability of the instrument in different cultural and linguistic contexts, as well as its potential utility within universal screening programmes for neurodevelopmental disorders. The importance of these future lines of research is closely linked to the evolving nature of sociocognitive skills and the need for reliable tools capable of identifying developmental risk at the earliest possible stages.

The development of age-based normative data is equally important. The establishment of normative developmental curves would enable clinicians and researchers to distinguish between typical developmental variability and clinically significant developmental delays. Such normative references would also enhance the practical applicability of the ESCI in paediatric and educational contexts, facilitating early referral to specialist assessment and intervention services.

Future cross-cultural and cross-linguistic studies are also highly relevant, as sociocognitive development occurs within social and cultural contexts that may influence parental perceptions, communicative interaction and developmental expectations. Although many sociocognitive skills appear to be universal, the ways in which parents interpret and describe these behaviours may vary across cultures. Consequently, the validation of the ESCI across different linguistic and cultural populations would strengthen its international applicability and contribute to the development of culturally sensitive developmental screening tools.

## 5. Conclusions

The ESCI may prove more useful as an early development screening tool than as a measure for older preschool children. From a clinical perspective, the ESCI offers several practical advantages:

The ESCI is based on parental reports and can be administered relatively quickly. This makes it particularly suitable for large-scale screening programmes in nurseries, paediatric settings and early intervention services. Parent-reported measures also provide valuable information on children’s behaviour in naturalistic everyday contexts, which cannot always be captured during structured clinical observation.

Despite the above reported, the results of this pilot study provide encouraging evidence regarding the psychometric properties and clinical utility of the Spanish adaptation of the ESCI. The instrument demonstrated adequate internal consistency, significant correlations with theoretically related constructs, and good diagnostic discrimination, particularly for autism spectrum disorder. These findings support the utility of the ESCI as a measure for the early detection of sociocognitive development.

## Figures and Tables

**Table 1:** Distribution of the sample by gender, age, and group affiliation.

	Neurodevelopmental Disorders (NDD)					Typical Development (TD)		
Age in Months	Language Disorders		Autism Spectrum Disorders					
	Male	Female	Male	Female	Total	Male	Female	Total
18–23	0	1	0	0	1	15	9	24
24–35	2	2	2	2	8	4	2	6
36–47	2	0	5	1	8	27	25	52
48–60	1	0	1	0	2	20	11	31
Total	5	3	8	3	19	66	47	113
	8		11			113		

**Table 2:** Descriptive statistics for the total ESCI score by age group in typical development children.

Age in Months	N	Mean	Standard Deviation	Minimum	Maximum
18 to 23	24	34.48	2.88	29.00	42.00
24 to 35	6	37.50	3.27	34.00	42.00
36 to 47	52	39.36	2.07	34.00	42.00
48 to 60	31	39.87	1.91	34.00	42.00
Total	113	38.33	3.10	29.00	42.00

**Table 3:** Correlation between the total ESCI score and the various scales used.

	Total Sample N = 132				Typical Development N = 113		ASD and Language Disorders N = 19	
	Total ESCI	*p*	Partial Correlation Age Control		Total ESCI	*p*	Total ESCI	*p*
PPVT	0.69	<0.001	0.58	<0.001	0.68	<0.001	0.40	0.099
ESB SR	0.55	<0.001	0.53	<0.001	0.25	0.008	0.51	0.032
ESB JA	0.66	<0.001	0.58	<0.001	0.48	<0.001	0.59	0.011
ESB SU	0.74	<0.001	0.64	<0.001	0.71	<0.001	0.54	0.022
ESB TOTAL	0.74	<0.001	0.66	<0.001	0.65	<0.001	0.58	0.012
AWR	−0.59	<0.001	−0.54	<0.001	−0.41	<0.001	−0.46	0.052
COG	−0.63	<0.001	−0.64	<0.001	−0.34	<0.001	−0.63	0.006
COM	−0.67	<0.001	−0.71	<0.001	−0.32	<0.001	−0.78	<0.001
MOT	−0.55	<0.001	−0.53	<0.001	−0.30	0.001	−0.62	0.006
RRB	−0.48	<0.001	−0.51	<0.001	−0.11	0.240	−0.35	0.154
SCI	−0.74	<0.001	−0.72	<0.001	−0.42	<0.001	−0.77	<0.001
SRS 2	−0.68	<0.001	−0.68	<0.001	−0.38	<0.001	−0.73	<0.001

**Table 4:** Means and standard deviations for the three groups of children (typical development, language disorders, and autism spectrum disorders).

	Typical Development			Language Disorders			Autism Spectrum Disorders		
Age in Months	Mean	Standard Deviation	N	Mean	Standard Deviation	N	Mean	Standard Deviation	N
18 to 23	34.48	2.88	24	38.00	.	1	-	-	-
24 to 35	37.50	3.27	6	33.75	3.09	4	29.00	6.98	4
36 to 47	39.36	2.08	52	34.50	0.71	2	30.17	3.66	6
48 to 60	39.87	1.91	31	41.00	.	1	25.00	.	1
Total	38.33	3.10	113	35.37	3.38	8	29.27	4.86	11

**Table 5:** ROC curve results.

	AUC	Error	GINI	Youden	Exploratory Cut-off Point	Sensitivity	Specificity	PPV	NPV
Either of the two	0.87	0.05	0.75	0.62	35.00	0.79	0.79	0.95	0.38
Language Disorders	0.75	0.09	0.50	0.42	38.00	0.88	0.50	0.98	0.10
Autism Spectrum Disorders	0.94	0.03	0.88	0.70	32.00	0.73	0.56	0.97	0.62

AUC: Area under the curve; PPV: Positive Predictive Value; NPV: Negative Predictive Value.

## Data Availability

The data that support the findings of this study are available on request from the corresponding author. The data are not publicly available due to privacy restrictions.

## Supplementary Materials

The following supporting information can be downloaded at: https://www.mdpi.com/article/10.3390/children13070932/s1, Supplementary Materials S1: Response frequencies for the three response options across the 21 items by age group; Supplementary Materials S2: Means, standard deviations and item-total correlations for the scale, and the probability of significance.
