# Supplementary material for: Early Sociocognitive Development Among Spanish-Speaking Participants: A Preliminary Study Using the Early Social Cognition Inventory (ESCI)
Source: Children (Basel). 2026 Jul 16;13(7):932. doi: 10.3390/children13070932 (PMC13406478; doi:10.3390/children13070932)
Supplement: Supplementary file 1 [file children-13-00932-s001.zip › children-4390693-supplementary.pdf]

	Age in months											
	18 to 24			24 to 36			37 to 48			49 to 60		
Item	Yes	No	Nk/No	Yes	No	Nk/No	Yes	No	Nk/No	Yes	No	Nk/No
1	26	1	0	3	0	0	54	0	6	22	0	1
2	19	2	6	3	0	0	56	0	4	23	0	0
3	27	0	0	3	0	0	60	0	0	23	0	0
4	18	2	7	3	0	0	59	0	1	23	0	0
5	8	1	18	2	0	1	33	0	27	17	0	6
6	18	0	9	2	0	1	60	0	0	22	0	1
7	25	2	0	3	0	0	50	0	10	21	0	2
8	26	0	1	3	0	0	59	0	1	23	0	0
9	18	1	8	3	0	0	36	0	24	13	0	10
10	8	1	18	2	0	1	32	0	28	17	0	6
11	27	0	0	3	0	0	58	0	2	21	0	2
12	3	2	22	1	0	3	41	0	19	17	0	6
13	22	0	5	3	0	0	58	0	2	23	0	0
14	28	1	0	3	0	0	56	0	4	22	0	1
15	4	3	20	1	0	2	57	0	3	21	0	2
16	25	1	1	3	0	0	57	0	3	23	0	0
17	27	0	0	3	0	0	60	0	0	22	0	1
18	3	3	21	1	0	2	47	0	13	19	0	4
19	8	3	16	1	0	2	52	0	8	22	0	1
20	14	2	11	2	0	1	57	0	3	22	0	1
21	16	3	8	3	0	0	59	0	1	23	0	0

Supplementary Materials S1: Response frequencies for the three response options across the 21 items by age group.

Item	Mean	Standard Deviation	r_{xy}	p
1	1.93	0.26	0.35	<0.001
2	1.89	0.31	0.65	<0.001
3	2	0.00	0.55	<0.001
4	1.91	0.29	0.64	<0.001
5	1.53	0.50	0.54	<0.001
6	1.90	0.30	0.40	<0.001
7	1.88	0.33	0.13	0.144
8	1.98	0.13	0.50	<0.001
9	1.62	0.49	0.21	0.014
10	1.52	0.50	0.58	<0.001
11	1.96	0.19	0.22	0.010
12	1.55	0.50	0.72	<0.001
13	1.94	0.24	0.61	<0.001
14	1.95	0.23	0.33	<0.001
15	1.73	0.44	0.74	<0.001
16	1.96	0.21	0.44	<0.001
17	1.99	0.09	0.52	<0.001
18	1.62	0.49	0.73	<0.001
19	1.73	0.44	0.67	<0.001
20	1.84	0.37	0.73	<0.001
21	1.89	0.31	0.73	<0.001

Supplementary Materials S2: Means, standard deviations and item-total correlations for the scale, and the probability of significance.
